# White matter hyperintensities in cholinergic pathways are associated with dementia severity in e4 carriers but not in non-carriers

**DOI:** 10.3389/fneur.2023.1100322

**Published:** 2023-02-14

**Authors:** Ming-Chun Yu, Yi-Fang Chuang, Shu-Ching Wu, Cheng-Feng Ho, Yi-Chien Liu, Chia-Ju Chou

**Affiliations:** ^1^Department of Neurology, Cardinal Tien Hospital, New Taipei City, Taiwan; ^2^Institute of Public Health, National Yang-Ming Chiao Tung University, Taipei, Taiwan; ^3^Department of Radiology, Cardinal Tien Hospital, New Taipei City, Taiwan; ^4^Medical School of Fu-Jen University, New Taipei City, Taiwan; ^5^Geriatric Behavioral Neurology Project, Tohoku University New Industry Hatchery Center (NICHe), Sendai, Japan

**Keywords:** mild cognitive impairment, Alzheimer's disease, white matter hyperintensities (WMHs), Cholinergic Pathways Hyperintensities Scale (CHIPS), apolipoprotein E (APOE)

## Abstract

**Background and objectives:**

Among individuals with Alzheimer's disease (AD), *APOE* e4 carriers with increased white matter hyperintensities (WMHs) may selectively be at increased risk of cognitive impairment. Given that the cholinergic system plays a crucial role in cognitive impairment, this study aimed to identify how *APOE* status modulates the associations between dementia severity and white matter hyperintensities in cholinergic pathways.

**Methods:**

From 2018 to 2022, we recruited participants (*APOE* e4 carriers, *n* = 49; non-carriers, *n* = 117) from the memory clinic of Cardinal Tien Hospital, Taipei, Taiwan. Participants underwent brain MRI, neuropsychological testing, and *APOE* genotyping. In this study, we applied the visual rating scale of the Cholinergic Pathways Hyperintensities Scale (CHIPS) to evaluate WMHs in cholinergic pathways compared with the Fazekas scale. Multiple regression was used to assess the influence of CHIPS score and *APOE* carrier status on dementia severity based on Clinical Dementia Rating—Sum of Boxes (CDR-SB).

**Results:**

After adjusting for age, education and sex, higher CHIPS scores tended to be associated with higher CDR-SB in *APOE* e4 carriers but not in the non-carrier group.

**Conclusions:**

Carriers and non-carriers present distinct associations between dementia severity and WMHs in cholinergic pathways. In *APOE* e4 carriers, increased white matter in cholinergic pathways are associated with greater dementia severity. In non-carriers, WMHs exhibit less predictive roles for clinical dementia severity. WMHs on the cholinergic pathway may have a different impact on *APOE* e4 carriers vs. non-carriers.

## 1. Introduction

Sporadic Alzheimer's disease (AD) is the most common cause of neurodegenerative diseases in older adults, accounting for ~10% of cases in the population above the age of 65 years in the US (1) and 7–8% of the same population worldwide, with a considerable growing prevalence noted in the Asia Pacific region (2). The progressive loss of cholinergic neurons in the basal forebrain (3, 4) is associated with cognitive impairment in AD dementia. Current evidence indicates that the widely distributed acetylcholine in the central nervous system plays a critical role in modulating cognitive function and memory processes (5, 6). The reduction in cholinergic receptor density or binding affinity was more significant in older adults with mild cognitive impairment (MCI) and AD dementia (7) compared with cognitively unimpaired older adults. The complex molecular interaction of the cholinergic system, amyloid precursor protein, and proinflammatory cytokines modulates the formation of β-amyloid plaques and deposition, contributing more to the pathogenesis of Alzheimer's disease (8).

White matter hyperintensities (WMHs) in cholinergic pathways can also impair the integrity of the cholinergic network (9). WMHs are the most noticeable and easily accessible marker of small vessel diseases and cerebral amyloid angiopathy on MRI (10). The prevalence of WMH is highly associated with age and AD pathologies. A more severe WMH burden is associated with worse cognitive function, although this association weakened while controlling for age and other risk factors (11). Additionally, few data have demonstrated that greater dementia severity is associated with higher WMH burdens in cholinergic pathways compared with the whole brain (12, 13).

Among risk factors for AD, such as age, sex, and education, the *APOE* e4 allele is the strongest known genetic risk factor for Alzheimer's disease despite unclear neurostructural substrates (14, 15). Both heterozygous and homozygous e4 alleles increase the risk for AD dementia by 3–6- and 8–10-fold, respectively (16). The apolipoprotein e4 allele is associated with greater burdens of amyloid deposition (17), cerebral microbleeds (18), and white matter hyperintensities (19–21). Previous studies indicated that numerous factors may interact with *APOE* e4 synergically to facilitate the progression of WMH (22). The WMH and *APOE* e4 alleles may not directly influence each other but both contribute to cognitive impairment, especially memory loss, in AD dementia. An interactive effect of WMH and the *APOE* e4 gene on clinical cognitive impairment has been reported by some studies (23); however, the exact mechanism or etiology remains unclear. By analyzing the association between WMH in the cholinergic pathway and dementia severity among *APOE* e4 carriers and non-carriers primarily with memory problems, our goal is to examine the potential alterations on WMH that e4 alleles might contribute to in the formation of AD dementia syndrome.

In this study, we adopted and focused on the Cholinergic Pathways Hyperintensities Scale (CHIPS) (13, 24) to evaluate the degree of WMH in cholinergic pathways. As visual rating method counterparts, besides CHIPS, we also applied the Fazekas scale and medial temporal lobe atrophy (MTA) as predictive variables for dementia severity. In this study, we examined the association among three visual rating scores and dementia severity in *APOE* e4 carriers and non-carriers. We hypothesize that greater WMHs in cholinergic pathways would be associated with greater dementia severity and that the APOE e4 gene plays a facilitating role in the increased severity of clinical dementia.

## 2. Methods

### 2.1. Participants

From January 2018 to July 2022, patients aged 50–90 years with either subjective or objective memory impairment for more than 6 months were recruited from the memory clinic in Cardinal Tien Hospital, Taipei, Taiwan. Participants received brain MRI, completed standardized neuropsychological tests, and *APOE* genotyping. Age, years of education, and diagnoses were documented. After medical evaluation and laboratory testing, participants with any possible reversible causes of dementia were excluded, such as those with brain tumors, metabolic diseases, and psychiatric history. All participants provided written informed consent, and the study was approved by the Research Ethics Committee/Institutional Review Board of Cardinal Tien Hospital, Taipei, Taiwan (CTH-110-2-1-014).

### 2.2. Neuropsychological tests

In this study, to evaluate general objective cognitive function, a unified standardized Mandarin Chinese version of the Mini-Mental Status Examination (MMSE) (25) was performed on each patient during the first clinic encounter. MMSE served as a baseline cognitive evaluation for the diagnosis of dementia.

The functional severity of dementia was evaluated by the global Clinical Dementia Rating (CDR) scale and the Clinical Dementia Rating scale Sum of Boxes (CDR-SB). The evaluation of CDR was based on five levels (0, 0.5, 1, 2, and 3) in six domains (memory, orientation, judgment, problem solving, community affairs, home and hobbies, and personal care). The CDR-SB score was calculated by adding six domains of functioning scores.

Compared to MMSE, CDR-SB incorporates evaluations on the domain of self-care, social impairment, and daily function, and is better at detecting prodromal dementia (26). As opposed to global CDR, CDR-SB provides a quantitative evaluation of impairment and can be treated as interval data in statistical analysis rather than the global CDR scale (27). In this study, CDR-SB is the primary outcome variable that we analyzed for dementia severity. All clinical information was provided by the caregivers and occasionally the patients themselves at the first clinic encounter to avoid the influence of medical treatment.

### 2.3. Diagnosis of dementia

Participants with subjective or objective memory impairment for more than 6 months were recruited from the memory clinic using the 2011 National Institute on Aging—Alzheimer's Association (NIA-AA) criteria (28). Participants with a global CDR scale of 0 are classified as normal participants; subjects with a CDR score of 0.5 are classified as having mild cognitive impairment (MCI); and subjects with a CDR score of 1 or 2 are diagnosed with probable Alzheimer's disease.

### 2.4. Brain MRI acquisition and visual rating scale in our study

All participants received brain MRI (GE, 3T DISCOVERY 750, GE Taiwan) with trans-axial T2 weighted scans, and 3D fluid-attenuated inversion recovery images and high-resolution sagittal T1-weighted images were obtained. We minimized the variations in MRI machines by using the same MRI machine at Cardinal Tien Hospital during participant enrollment. The image analysis included three visual rating methods that are described as follows.

### 2.5. Evaluation of white matter hyperintensities

The periventricular white matter hyperintensities measured based on the Fazekas scale were graded and evaluated by the author in T2-FLAIR axial view (29, 30). The periventricular Fazekas score (PV Fazekas) was rated on a 4-point scale as follows: 0, absent; 1, mild; 2, moderate; and 3, severe; in addition, deep white matter Fazekas score (DWM) was evaluated on the same 4-point scale as an alternative to white matter hyperintensity visual rating scores.

### 2.6. Evaluation of cholinergic pathway hyperintensities scale

**Figure 1:**
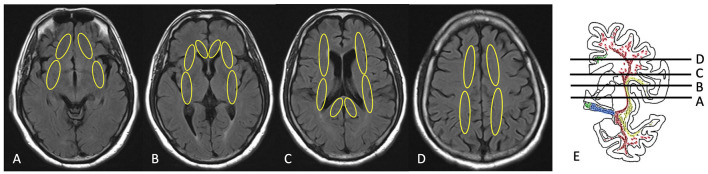
Illustration of CHIPS scoring on brain MRI. **(A)** Low external capsule, **(B)** high external capsule and anterior cingulate gyrus, **(C)** corona radiata and posterior cingulate gyrus, **(D)** centrum semiovale, **(E)** coronal view [immunohistochemical tracings of the cholinergic pathways with levels for selected slices **(A–D)** presented in the axial plane; drawing from Selden (31)].

White matter hyperintensities in cholinergic pathways were graded visually using the Cholinergic Pathways HyperIntensity Scale (CHIPS) by the first author on a single-rater basis. First reported by Bocti et al. (24), CHIPS is a visual rating scale developed based on published immunohistochemical tracings of the cholinergic pathways in humans (24, 31), and was previously used by some studies for the relationship between WMH and cognition (12, 13). According to Selden (31), the cholinergic pathways include the medial pathway and lateral pathway. The medial pathway is closely associated with the adjacent cingulate gyrus and rostrum of the corpus callosum; the lateral pathway courses through the external capsule and claustrum within the white matter (31). Accordingly, four axial planes of T2-FLAIR images were identified by major anatomical landmarks—low external capsule, high external capsule, corona radiata, and centrum semiovale (Figure 1). Medial pathway is included in two of the axial planes as anterior cingulate gyrus and posterior cingulate gyrus. A total of 10 regions are illustrated in Figures 1A–D. White matter hyperintensity of each region was determined visually on a 3-point scale for each region (0 = normal; 1 = minimal; 2 = confluent or moderate to severe). To account for the decreasing concentration of cholinergic fibers, each slice was weighted sequentially from 1 to 4 with one being the centrum semiovale and four being the lower external capsules (Table 1). The total CHIPS score (both hemispheres) ranged from 1 to 100. The lowest CHIPS score is 0, indicating no burden of WMH in cholinergic pathways, and the highest CHIPS score is 100 (24). The corresponding author independently rated CHIPS scores of random 65 participants to ascertain the inter-rater reliability of CHIPS. Controversial images were rated based on the consensus of the first author, the corresponding author, and a radiologist (Cheng-Feng Ho). The consensus CHIPS scores were used in our regression analyses. Intra-rater reliability was calculated by two independent ratings of the first author. The inter-rater reliability and intra-rater reliability were analyzed by inter-class correlation coefficient (ICC).

**Table 1:** The Cholinergic Pathways Hyperintensities Scale (CHIPS) evaluation of unilateral hemisphere.

**Axial planes of T2-FLAIR**	**Regions**	Score*	**Weighting factor**	**Total**
Low external capsule	Anterior EC	0–1–2	4	0–4–8
	Posterior EC	0–1–2	4	0–4–8
High external capsule	Cingulate	0–1–2	4	0–4–8
	Anterior EC	0–1–2	3	0–3–6
	Posterior EC	0–1–2	3	0–3–6
Corona radiata	Anterior EC	0–1–2	2	0–2–4
	Posterior EC	0–1–2	2	0–2–4
	Cingulate	0–1–2	1	0–1–2
Centrum semiovale	Anterior EC	0–1–2	1	0–1–2
	Posterior EC	0–1–2	1	0–1–2

EC, external capsule.

^*^0, normal; 1, minimal; 2, confluent or moderate to severe.

### 2.7. Evaluation of medial temporal lobe atrophy

Medial temporal lobe atrophy, also known as the Scheltens' scale, is a sign of neurodegenerative disease and a strong predictor of clinical cognitive impairment (32, 33). MTA was evaluated by the first author in the coronal cut through T1-weighted images, and was rated on a 5-point scale based on the height of the hippocampal formation and the width of the choroid fissure and the temporal horn as follows: 0, absent; 1, minimal; 2, mild; 3, moderate; and 4, severe (32). The MTA score was applied to both the right and left medial temporal lobes, separately. In our study, both sides were summed to determine the degree of atrophy.

### 2.8. Statistical analyses

All analyses were performed using STATA version 16 (College Station, TX, USA). Among the two groups, including carriers and non-carriers, demographic variables were compared using two-tailed Student's *t*-test for continuous variables, the chi-square independent test for categorical variables, and Fisher's exact test as appropriate. Pearson's correlations were analyzed between different visual rating scores. An alpha of 0.05 was set for statistical significance. General cognition was measured using the MMSE. The MMSE score and CDR-SB were used to compare cognition in *APOE* e4 carriers and non-carriers at baseline. For analyses, we used CDR-SB as the primary outcome variable due to its detailed quantitative nature for dementia severity scores. Additional regression analyses using MMSE as an outcome variable were performed as a comparison to CDR-SB.

First, we tested associations between visual rating scores and dementia severity using simple linear regression in *APOE* e4 carriers and non-carriers, separately. Second, multiple well-studied dementia predictors, such as age and years of education, were used to assess the effects of WMH in cholinergic pathways on CDR-SB scores. We performed multiple linear regressions for CHIPS, Fazekas scale, and MTA in separate regression models, further including their interactions with *APOE* e4 status as predictors. In previous literature, more evidence suggests that periventricular white matter lesions, rather than deep white matter hyperintensities, are associated with cognitive impairment (34). We choose periventricular Fazekas scale as a visual rating scale counterpart for CHIPS due to their anatomical overlaps and higher association with cognitive performance. CDR-SB was the primary outcome variable. In all models, age, sex, and education were included as predictor variables. Regression Model 0 exclusively includes age, sex, and education as predictive factors. Due to the high correlation between visual rating scores, CHIPS, MTA, and the Fazekas scale were separately added to Model 0 to decrease statistical collinearity. In Model 1, CHIPS and CHIPS^*^e4 status were included as predictors. Model 2 included the Fazekas scale and Fazekas^*^e4 status as predictors. Model 3 included the MTA scores and MTA^*^e4 status as predictors. In Model 4, we included CHIPS scores, CHIPS^*^e4 status, and MTA scores despite the potential collinearity of CHIPS and MTA, for MTA and CHIPS are two distinctive visual rating measurements anatomically and etiologically. Third, additional multiple linear regression models using MMSE as the outcome variable were analyzed in a similar manner with predictive variables (CHIPS, PV Fazekas, MTA) respectively added into models.

## 3. Results

### 3.1. Demographic data and image evaluation

The demographic data of *APOE* e4 carriers and non-carriers are presented in Table 2. Of the 166 subjects, 49 were *APOE* e4 carriers (29.5%), and 117 were non-carriers (70.5%). Among the 49 e4 carriers, 44 had the e3e4 genotype, 3 had the e4e4 genotype, and 2 had the e2e4 genotype. The diagnoses included Alzheimer's disease (AD, 22.891%, *n* = 38), mild cognitive impairment (MCI, 47.590%, *n* = 79), and normal (29.518%, *n* = 49). In ADs, 19 subjects were e4 carriers, and 19 subjects were non-carriers. For MCIs, 19 subjects were e4 carriers, and 62 subjects were non-carriers. In total, 13 normal participants were carriers, and 36 were non-carriers. Fisher's exact test revealed an association between participants' diagnoses and the *APOE* e4 allele (Fisher's exact test *p*-value = 0.008). Age, sex, and years of education did not differ between *APOE* e4 carriers and non-carriers. The average MMSE of the carriers was significantly lower than that of the non-carriers (Student's *t*-test *p*-value = 0.003). Regarding global CDR scores, the numbers of *APOE* e4 carriers and non-carriers differed significantly (Fisher's exact test *p*-value = 0.009). Higher CDR-SB scores were observed in carriers than in non-carriers (Student's *t*-test *p*-value < 0.001). Regarding visual rating scores, the mean periventricular Fazekas scores were significantly lower in carriers (Student's *t*-test *p*-value = 0.038); carriers also have a lower mean of deep white matter Fazekas scale without statistical significance (Student's *t*-test *p*-value = 0.232). Neither CHIPS nor MTA scores exhibited between-group differences. In all participants, there is a strong correlation between periventricular Fazekas scale and deep white matter Fazekas scale (Pearson's *r* = 0.693, *p*-value < 0.001). Either periventricular or DWM Fazekas scale presented high correlations with CHIPS. There is a small to mediate correlation between CHIPS and MTA in our participants (Pearson's *r* = 0.285, *p*-value < 0.001). For total CHIPS scores, the inter-rater reliability (ICC = 0.934, 95% confidence interval 0.894 < ICC < 0.959) and intra-rater reliability (ICC = 0.985, 95% confidence interval 0.975 < ICC < 0.991) were both high.

**Table 2:** Demographic and clinical data in two groups of study participants defined as carriers and non-carriers based on *APOE* e4 genotype.

	**Carriers** ***n* = 49 (29.5%)**	**Non-carriers** ***n* = 117 (70.5%)**	***p*-value**
Age (yrs)	76.7 ± 7.6	76.4 ± 7.7	0.807
Female (*n*, %)	31 (63.3%)	70 (59.8%)	0.679
Education (yrs)	9.4 ± 5.4	9.7 ± 4.8	0.712
MMSE	19.7 ± 8.1	23.3 ± 6.3	0.003
CDR (*n*, %)			0.009
0	13 (7.8%)	36 (21.7%)	
0.5	17 (10.2%)	62 (37.4%)	
1	13 (7.8%)	16 (9.6%)	
2	6 (3.6%)	3 (1.8%)	
CDR-SB	3.9 ± 3.8	2.1 ± 2.8	< 0.001
PV Fazekas	1.4 ± 0.8	1.8 ± 1.0	0.038
DWM Fazekas	1.3 ± 0.1	1.5 ± 0.1	0.232
MTA	2.6 ± 1.6	2.5 ± 1.8	0.611
CHIPS	15.6 ± 11.4	15.0 ± 12.6	0.789

MMSE, Mini-Mental State Examination; CDR, Clinical Dementia Rating Scale; CDR-SB, Clinical Dementia Rating Scale-Sum of Boxes scores; PV Fazekas, periventricular Fazekas Scale; DWM Fazekas, deep white matter Fazekas Scale; MTA, Medial Temporal Atrophy; CHIPS, Cholinergic Pathways Hyperintensities Scale.

The *p*-values represent the results of the comparison between APOE e4 carriers vs. e4 non-carriers. Values are the mean ± standard deviation and number (%).

### 3.2. Results of simple linear regression and multiple linear regression analyses

**Figure 2:**
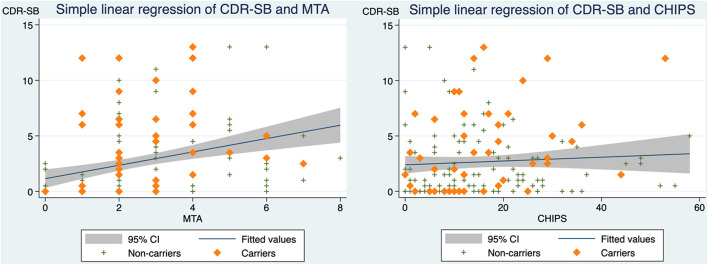
The correlations between CDR-SB and MTA as well as CDR-SB and CHIPS in all participants. **(Left)** Scatterplot depicting CDR-SB scores and MTA with a simple linear regression line and 95% confidence interval (standardized beta coefficient = 0.322, *R*^2^ = 0.104, adjusted *R*^2^ = 0.098, *p*-value < 0.001). **(Right)** Scatterplot depicting CDR-SB scores and CHIPS scores with a simple linear regression line and 95% confidence interval (standardized beta coefficient = 0.066, *R*^2^ = 0.004, adjusted *R*^2^ = −0.002, *p*-value = 0.397). CDR-SB, clinical dementia rating scale-sum of boxes scores; MTA, medial temporal atrophy; CHIPS, Cholinergic Pathways Hyperintensities Scale.

**Figure 3:**
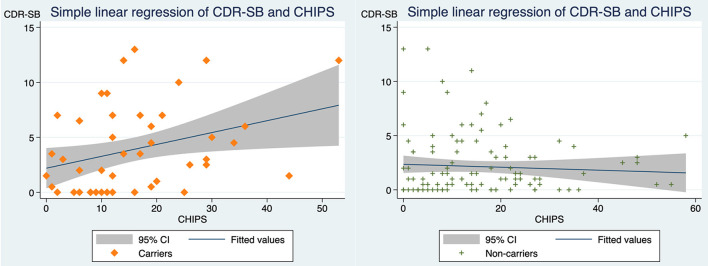
The correlations between CDR-SB and CHIPS in *APOE*4 carriers vs. non-carriers. **(Left)** In carriers, scatterplot depicting CDR-SB scores and CHIPS scores with a simple linear regression line and 95% confidence interval (standardized beta coefficient = 0.319, *R*^2^ = 0.101, adjusted *R*^2^ = 0.082, *p*-value = 0.026). **(Right)** In non-carriers, scatterplot depicting CDR-SB scores and CHIPS with simple linear regression line and 95% confidence interval (standardized beta coefficient = −0.062, *R*^2^ = 0.004, adjusted *R*^2^ = −0.005, *p*-value = 0.509). CDR-SB, clinical dementia rating scale-sum of boxes scores; CHIPS, Cholinergic Pathways Hyperintensities Scale; Carriers, APOE e4 heterozygous or homozygous carriers; Non-carriers, APOE e4 non-carriers.

Our findings of correlations for all participants indicated that CDR-SB correlates with MTA (standardized beta coefficient = 0.322, *p*-value < 0.001) and that CDR-SB is not correlated with CHIPS (Figure 2). However, when assessing CHIPS and CDR-SB separately in the *APOE* e4 carrier group and the non-carrier group, two distinct correlations are noted (Figure 3). CHIPS scores were associated with CDR-SB scores (*p*-value = 0.027) in the carriers but not in non-carriers. No significant associations between Fazekas scores and CDR-SB scores were observed in either carriers (*p*-value = 0.409) or non-carriers (*p*-value = 0.739).

Multiple linear regression analyses, using CDR-SB as the outcome variable, revealed that older age was a significant predictive factor in Models 0, 1, 2, 3, and 4 (Table 3). Model 1 analysis indicated that CHIPS was not significantly associated with CDR-SB, but an interaction of *APOE* e4 on CHIPS was demonstrated to be significant (*p*-value = 0.044). In Model 1, a greater CHIPS score was associated with higher CDR-SB in *APOE* e4 carriers only (beta = 0.064, *p*-value = 0.097); no such associations were observed in non-carriers (beta = −0.024, *p*-value = 0.270). In Model 2, the Fazekas scale showed no significance in prediction and no interaction with *APOE* e4. Model 3 indicated that the MTA score was significantly predictive of CDR-SB in all participants (*p*-value = 0.008) without an interactive effect between the MTA and e4 alleles (*p*-value = 0.427). In Model 4, where both CHIPS and MTA were included into regression analysis, the significant predictive factors are age, MTA, and the *APOE* e4 interactions on CHIPS (*p*-value = 0.043).

**Table 3:** Multiple linear regression models of CDR-SB as the outcome variable.

**CDR-SB**	**Model 0**				**Model 1—CHIPS**				**Model 2—Fazekas**				**Model 3—MTA**				**Model 4—CHIPS and MTA**			
**Pre- dictors**	**Beta (standa-** **rdized)**	**SE**	***T***	***p***	**Beta (standa-** **rdized)**	**SE**	***T***	***p***	**Beta (standa-** **rdized)**	**SE**	***T***	***p***	**Beta (standa-** **rdized)**	**SE**	***T***	***p***	**Beta (standa-** **rdized)**	**SE**	***T***	***p***
Intercept	−4.081	2.669	−1.530	0.128	−4.093	2.577	−1.590	0.114	−4.653	2.627	−1.770	0.078	−3.839	2.518	−1.520	0.129	−3.041	2.500	−1.220	0.226
Age	0.099 (0.238)	0.032	3.050	0.003	0.095 (0.228)	0.031	3.020	0.003	0.104 (0.250)	0.033	3.180	0.002	0.072 (0.174)	0.032	2.280	0.024	0.067 (0.161)	0.031	2.140	0.034
Sex	−0.474 (−0.116)	0.505	−0.940	0.349	−0.362 (−0.056)	0.486	−0.750	0.457	−0.446 (−0.069)	0.493	−0.900	0.367	−0.662 (−0.102)	0.476	−1.390	0.166	−0.609 (−0.094)	0.474	−1.290	0.200
Education	−0.074 (−0.106)	0.052	−1.430	0.153	−0.063 (−0.099)	0.050	−1.260	0.209	−0.075 (−0.117)	0.050	−1.490	0.138	−0.042 (−0.066)	0.049	−0.860	0.393	−0.045 (−0.071)	0.049	−0.930	0.355
e4					0.468 (0.067)	0.836	0.560	0.576	1.505 (0.215)	0.997	1.510	0.133	1.176 (1.168)	0.914	1.290	0.200	0.480 (0.069)	0.805	0.600	0.552
CHIPS					−0.024 (−0.093)	0.022	−1.110	0.270									−0.041 (−0.156)	0.021	−1.890	0.060
CHIPS*e4					0.088 (0.261)	0.043	2.030	0.044									0.086 (0.253)	0.042	2.040	0.043
Fazekas									−0.204 (−0.063)	0.279	−0.730	0.466								
Fazekas*e4									0.178 (0.043)	0.582	0.300	0.761								
MTA													0.414 (0.226)	0.155	2.670	0.008	0.505 (0.275)	0.139	3.630	< 0.001
MTA*e4													0.243 (0.110)	0.304	0.800	0.427			
Observations	166				166				166				166				166			
R/R adjusted	0.098/0.081				0.188/0.157				0.169/0.138				0.228/0.199				0.250/0.217			
Probability	*p* < 0.001				*p* < 0.001				*p* < 0.001				*p* < 0.001				*p* < 0.001			
Homoske- dasticity White's test	Chi squares = 8.540				Chi squares = 29.770				Chi squares = 33.910				Chi squares = 36.360				Chi squares = 58.860			
	*p* = 0.383				*p* = 0.124				*p* = 0.050				*p* = 0.028				*p* = 0.001			
	Residuals				Residuals				Residuals				Residuals				Residuals			
Skewness	1.524				1.393				1.456				1.495				1.281			
Kurtosis	5.254				5.357				5.603				5.372				4.702			

Beta, beta co-efficient; SE, standard error; T, statistics; *p*, p value; CDR-SB, Clinical Dementia Rating Scale Sum of Boxes scores; CHIPS, Cholinergic Pathway Hyperintensity Scale; CHIPS^*^e4, The interactive effect of the APOE e4 gene on cholinergic pathway hyperintensity; Fazekas, periventricular Fazekas Scale; Fazekas^*^e4, The interactive effect of the APOE e4 gene on the periventricular Fazekas scale; MTA, Medial Temporal Atrophy; MTA^*^e4, The interactive effect of the APOE e4 gene on Medial Temporal Atrophy.

Multiple linear regression analyses, using MMSE as the outcome variable, suggested that age and education were significantly predictive in all models. In the model that includes MTA, MTA demonstrated significant predictive effect for MMSE. There was no *APOE* e4 interactions in any of the models using MMSE as the outcome variable. Neither CHIPS nor Fazekas scale had predictive power for MMSE.

## 4. Discussion

This is a cross-sectional study comparing the association between white matter hyperintensities in cholinergic pathways and the severity of dementia among two groups, namely, apolipoprotein e4 carriers and non-carriers. Our findings suggest two distinct associations between WMHs and clinical dementia severity across carriers and non-carriers. Under the context of a similar demographic background, the e4 carriers presented lower MMSE and higher CDR-SB scores, which is consistent with previous literature indicating that the e4 phenotype is associated with greater dementia severity (35–37). Other visual rating scores (CHIPS, MTA score, periventricular Fazekas scale, and DWM Fazekas scale) were analyzed by correlation and regression models. This finding suggests that CDR-SB correlates with WHMs in cholinergic pathways exclusively in e4 carriers and not in non-carriers. An interaction for the *APOE* e4 gene between dementia severity and CHIPS was noted.

The average CHIPS values of the two study groups were not significantly different, indicating that the degree of white matter changes in cholinergic pathways is visually similar among carriers and non-carriers. CHIPS alone does not demonstrate a clear and significant association with dementia severity (CDR-SB) as the MTA score does. Next, we separated participants into e4 carriers and non-carriers. We noticed that it is only in the carriers that present significant correlations between WMHs in cholinergic pathway and dementia severity as depicted in Figure 3. After adjusting for age, sex, and education in our regression models, CHIPS is only predictive for CDR-SB with the facilitating role of *APOE* e4. Even when MTA was added as an additional predictive variable, the e4 interaction is still significant for CHIPS and CDR-SB. Namely, for carriers, more WMH burden in cholinergic pathway associates with worse CDR-SB. CHIPS has no predictive role for CDR-SB for non-carriers. A similar result on WMHs was demonstrated by Mirza et al. (23), where general WMH volumes were associated with worse cognitive performance exclusively in *APOE* e4 carriers but not in non-carriers. Apolipoprotein E is one of the primary apolipoproteins in the lipid metabolism of the central nervous system (CNS) (38). Its role in lipid homeostasis and its anti-inflammatory effects have been investigated and supported for decades (17). Neuroinflammation is one of the key pathogenic factors in Alzheimer's disease (39). In e4 carriers, the increased level of inflammation may cause more covert damage to the neurons and nerve tracts, leading to earlier or worse dementia presentation (23, 40, 41).

In our analysis, both periventricular Fazekas and deep white matter Fazekas were correlated with CHIPS. Periventricular Fazekas score is the predictive variable we used in our regression models, yet in our analyses, neither periventricular Fazekas scale nor DWM Fazekas scale had predictive power for clinical dementia severity in either group. The Fazekas scale provides a general estimation of white matter change by grading the brain as a whole entity by a scale of 0–3 (30), but it does not compare or separate cerebral regions based on their functionality. This may limit its clinical utility due to non-specificity. Of note, in our analysis, the non-carrier group with better cognitive performance exhibits higher Fazekas scores. This finding suggests that WHMs in specific pathways instead of WMHs in general may be a better indicator of clinical dementia severity (42). In addition to the Fazekas scale, another commonly used WMH visual measurement, the Age-Related White Matter Change (ARWMC) score, has not been routinely applied as a clinical predictor for clinical dementia severity. This scale separates both the left and right hemispheres into five anatomical regions with a total sum-up score of 0–30 (43, 44). Although ARWMCs are easy and more detailed to apply to radiological images, this score still separates brain regions anatomically instead of functionally.

It is current knowledge that white matter changes are etiologically heterogeneous but present similar signals under brain magnetic resonance (10). Hyperintensities on MRI T2 FLAIR represent subtle water content differences from the “non-hyperintense” regions, which can result from different etiologies, such as small vessel diseases, microbleeds, ischemia, axonal loss, or demyelination (16). Over decades, measurements of white matter lesions have evolved from visual rating methods to semi-automated volumetry to tractography. Some evidence suggests high reliability and more efficacy of semi-automatic volume measurement than visual rating methods (45, 46). We used visual rating scale in this study, CHIPS in particular, which was validated by high correlation to the volumetric analysis of the cholinergic pathways (24). CHIPS evaluates hyperintensity signals in cholinergic pathways within the white matter. Previous DTI investigation substantiated the correlation between cognitive impairment and volume reduction or integrity reduction of cholinergic tracts (47–49). However, evidence of nerve tract atrophy or damage can not necessarily be interpreted as definite white matter signal changes or vice versa. The fact that carriers and non-carriers present similar CHIPS scores but only carriers demonstrate stronger clinical correlation further corroborates the heterogeneity of WMH and shed a light on possible alterations in WMH formation caused by e4 alleles.

Based on different models of multiple linear regression, MTA was identified as a significant independent variable for dementia severity in both carriers and non-carriers. The e4 interaction with MTA was not observed in the MTA models. Medial temporal lobe atrophy has long been demonstrated to be associated with amnestic cognitive impairment, and its correlation to Alzheimer's disease severity is substantial (32, 33). Nonetheless, dementia results from a multifactorial process. It is also known that MTA per se is insufficient to predict Alzheimer's pathologic change (50). In addition to neurodegeneration, other factors also contribute to clinical cognitive impairment, such as years of education, hearing impairment, and *APOE* genotype (51). The findings of this study suggest different pathogenicities for clinical dementia severity across e4 carriers and non-carriers. Regardless of neurodegeneration, we assume that the involvement of the e4 gene alters the formation of WMHs. With the e4 alleles conduces worse lipid metabolism and increased CNS inflammation, white matter lesions are more detrimental and destructive to neurons in carriers. Consequently, the destruction of cholinergic pathways manifests clinically as greater dementia severity. In our study population, two primary distinct pathoetiologies of WMH formation are proposed. For carriers, white matter lesions may, to a greater degree, be derived from neuronal inflammation, lipid metabolic dysfunction, and amyloid angiopathy; for non-carriers, WMHs could be the result of aging.

One of the limitations of this study involves the use of a visual rating method for WMHs. Despite high inter-/intra-rater reliability and time-saving characteristics, visual measurement in neuroimaging is still subjective and qualitative in nature. Some studies have indicated that visual rating methods are less sensitive to WMH volume measurements (52). Although Bocti et al. (24), has demonstrated high correlation of CHIPS and semi-automatic volumetric measurement, more objective methods may be adopted for future studies. The application of semi-automatic WMH volume measuring in cholinergic pathways may be worth investigating across e4 carriers and non-carriers. Further analysis comparing DTI and white matter signals in cholinergic pathways, examining e4 status and clinical severity, would be valuable for the etiology in clinical dementia syndrome. Additional limitation of this study is the absence of biomarker information from our participants, such as CSF study, amyloid PET, and tau distribution. It is possible that a proportion of patients in the normal group and MCI group eventually reflect a non-AD pathology. Other limitations of this study include the cross-sectional study design and the clinic-based recruitment. A community-based longitudinal cohort study design may further examine the temporal and causal relationships with less selection bias. Further community-based cohort study to examine the associations between WMHs in cholinergic pathways and clinical dementia staging would be conducive.

## 5. Conclusions

This study demonstrates the presence of an *APOE* e4 gene interaction on the associations between dementia severity (CDR-SB) and WMHs in cholinergic pathways. More white matter lesions in cholinergic pathways correlate with greater dementia severity in e4 carriers but not in non-carriers. This finding supports the importance of the cholinergic system in cognitive performance, the heterogeneity of WMHs in neuroimaging, and the lipid homeostasis and anti-inflammatory effects of apolipoprotein e4. In e4 carriers, due to possible e4 alterations in WMH formation, it is possible that WMHs are more detrimental, so lesions on the cholinergic pathways present greater dementia severity. General WMH gradings that do not consider functionality, such as the Fazekas scale or ARWMC, may play a less significant role in clinical symptoms in subjects with MCI and AD. After adjusting for age and education, CHIPS was only predictive for CDR-SB under *APOE* e4 interaction. The role of the *APOE* e4 genotype in cognitive outcome requires further investigation.

## Data availability statement

The raw data supporting the conclusions of this article will be made available by the authors, without undue reservation.

## Ethics statement

The studies involving human participants were reviewed and approved by the Institutional Review Board of Cardinal Tien Hospital, Taipei, Taiwan (IRB: CTH-110-2-1-014). The patients were given a complete description of the study, and each patient signed an informed consent form. The patients/participants provided their written informed consent to participate in this study.

## Author contributions

M-CY, Y-FC, C-JC, and Y-CL contributed to manuscript writing and revision. M-CY and S-CW contributed to preparing and sorting the data. M-CY, Y-CL, and C-FH contributed to interpreting the neuroimages. All authors have read and approved the final manuscript.
