# Supplementary material for: White matter hyperintensities in cholinergic pathways are associated with dementia severity in e4 carriers but not in non-carriers
Source: Front Neurol. 2023 Feb 14;14:1100322. doi: 10.3389/fneur.2023.1100322 (PMC9971995; doi:10.3389/fneur.2023.1100322)
Supplement: Supplementary file 1 [file Data_Sheet_1.PDF]

subject number	age	Gender	years of education	InitialCDR	InitialMMSE	InitialCDRsb	CarrierYesNo	APOE	CHIPS	MTA	Fazekas_PV	F_DWM	StudyDx	CHIPS_Liu	CHIPS_YU2nd
2	68	male	9	1	17	6	Non-carriers	e3e3	0	1	0	1	AD	0	0
4	87	female	0	2	9	9	Carriers	e3e4	11	2	1	1	AD		
5	88	female	12	.5	20	5	Carriers	e2e4	19	3	2	2	AD		
9	73	female	12	.5	17	3	Carriers	e3e4	3	2	0	0	AD	3	3
11	87	female	5	2	6	12	Carriers	e3e4	29	1	3	2	AD		
12	78	male	9	.5	23	3	Carriers	e3e4	29	7	3	3	AD		
15	85	female	9	1	24	0	Non-carriers	e3e3	14	3	3	3	AD		
16	73	female	6	.5	27	1	Carriers	e3e4	19	1	1	1	MCI		
17	88	male	9	.5	26	1	Non-carriers	e3e3	27	1	3	2	MCI	27	
18	71	female	6	.5	18	5	Non-carriers	e2e3	12	2	2	2	AD		
20	80	male	12	0	28	1	Carriers	e3e4	4	2	0	1	MCI	1	6
21	74	male	9	0	24	0	Non-carriers	e3e3	3	1	0	1	MCI	3	3
22	76	female	9	.5	22	4	Non-carriers	e3e3	6	2	3	1	AD		
23	86	female	9	.5	29	1	Non-carriers	e3e3	28	2	3	3	MCI	31	
24	74	female	9	.5	26	1	Non-carriers	e3e3	28	2	3	3	AD		
25	84	male	0	.5	24	0	Non-carriers	e3e3	7	4	0	1	MCI	10	6
26	72	female	6	.5	15	1	Non-carriers	e3e3	24	2	2	2	AD	9	
27	77	female	12	.5	23	2	Non-carriers	e2e3	9	2	3	2	AD	6	
28	78	female	12	.5	28	1	Non-carriers	e3e3	2	2	1	0	MCI		2
29	73	female	0	.5	22	1	Non-carriers	e3e3	16	1	1	1	MCI		
30	82	female	12	.5	29	1	Non-carriers	e3e3	23	3	3	3	MCI	25	
31	82	male	12	.5	27	1	Non-carriers	e3e3	21	7	3	2	MCI		
32	77	female	6	.5	27	1	Non-carriers	e3e3	24	2	2	2	MCI		
33	82	female	12	.5	28	0	Non-carriers	e3e3	5	2	0	1	MCI		5
34	87	male	9	.5	27	1	Non-carriers	e3e3	13	6	3	2	MCI		
35	80	female	6	.5	28	1	Non-carriers	e3e3	20	2	2	1	MCI		
36	88	male	9	.5	19	4	Non-carriers	e3e3	4	3	2	1	AD	2	2
37	72	male	12	.5	18	2	Non-carriers	e2e2	9	2	1	3	MCI		
38	76	male	12	.5	24	3	Non-carriers	e3e3	26	5	3	3	AD		
39	80	male	9	.5	23	1	Carriers	e3e4	20	3	2	1	AD		
40	70	male	22	0	29	0	Carriers	e3e4	2	2	1	1	Normal	3	2
41	77	male	12	0	30	0	Carriers	e4e4	12	2	1	2	Normal		
43	89	male	6	.5	28	1	Non-carriers	e3e3	26	2	2	3	MCI		
44	80	female	12	0	28	0	Non-carriers	e3e3	3	1	2	0	Normal	19	3
45	78	female	12	0	30	0	Non-carriers	e2e3	19	1	2	2	Normal		
46	61	female	16	0	29	0	Non-carriers	e2e3	19	0	1	1	Normal		
47	66	male	22	1	19	9	Non-carriers	e3e3	0	3	0	1	AD		0
50	80	male	16	0	30	0	Non-carriers	e3e3	1	2	1	1	Normal		1

52	74	female	6	.5	13	2	Non-carriers	e3e3	15	4	2	2	MCI		
53	79	female	0	1	15	5	Carriers	e3e4	12	3	1	1	MCI		
55	83	female	12	1	18	7	Non-carriers	e3e3	16	2	0	0	AD		
58	86	female	3	1	7	5	Non-carriers	e3e3	58	7	3	3	AD		58
60	71	female	16	0	29	0	Non-carriers	e3e3	27	2	1	1	Normal		
61	72	female	16	0	30	0	Non-carriers	e3e3	0	0	0	1	Normal		0
62	71	male	12	0	29	0	Non-carriers	e2e3	27	2	2	2	MCI	7	
63	79	female	12	0	29	0	Non-carriers	e3e3	7	2	1	1	Normal	7	
65	76	male	9	.5	28	1	Non-carriers	e3e3	12	2	3	2	MCI		
66	82	female	12	.5	23	4	Non-carriers	e3e3	11	2	2	2	MCI		
67	83	male	3	1	13	5	Non-carriers	e2e3	15	2	3	3	AD	6	
68	71	female	12	0	28	0	Non-carriers	e3e3	8	1	1	2	Normal		
69	62	female	12	0	28	0	Carriers	e3e4	25	1	1	2	Normal		
71	70	male	6	.5	24	2	Non-carriers	e3e3	8	4	1	1	MCI	8	
72	68	male	12	0	29	0	Carriers	e3e4	11	3	2	1	Normal	13	
73	78	female	12	0	27	0	Non-carriers	e3e3	15	2	1	1	MCI	13	
75	68	male	14	.5	20	4	Carriers	e3e4	3	5	1	2	MCI	9	1
76	55	female	12	0	30	0	Carriers	e3e4	9	2	1	3	Normal		
77	72	female	16	.5	28	1	Non-carriers	e3e3	4	3	2	1	MCI		4
78	73	male	12	.5	23	1	Non-carriers	e2e3	12	6	1	1	MCI		
79	71	female	14	0	30	0	Non-carriers	e2e3	0	0	0	0	Normal		0
80	81	female	6	.5	14	4	Non-carriers	e3e3	2	2	2	1	MCI		2
83	71	male	14	.5	28	1	Non-carriers	e3e3	7	0	2	2	Normal	7	
84	82	female	6	0	29	0	Carriers	e3e4	8	1	1	1	MCI		
86	88	male	12	.5	26	3	Non-carriers	e3e3	27	3	3	2	MCI	27	27
87	76	male	12	0	29	0	Carriers	e3e4	16	0	1	2	MCI		
88	72	female	4	.5	14	5	Carriers	e3e4	33	6	2	3	AD		43
89	75	female	0	.5	22	1	Non-carriers	e3e3	9	2	1	1	MCI		
90	82	female	0	.5	20	4	Non-carriers	e3e3	18	5	1	1	AD		
91	83	male	6	.5	19	4	Carriers	e3e4	14	4	1	0	AD		
92	80	male	16	0	30	0	Non-carriers	e2e3	4	2	2	2	Normal		4
93	73	female	14	0	28	0	Non-carriers	e3e3	0	2	1	0	Normal	0	0
94	88	female	9	2	4	12	Carriers	e3e4	14	2	2	1	AD		
96	67	female	16	.5	24	2	Non-carriers	e2e3	9	2	2	2	MCI		
97	67	female	6	.5	16	5	Non-carriers	e3e3	11	3	1	1	AD	9	
99	76	male	18	0	30	0	Carriers	e3e4	5	0	1	0	Normal	5	5
100	85	female	0	.5	5	3	Carriers	e3e4	26	2	2	2	MCI		
102	81	female	6	1	15	7	Non-carriers	e3e3	22	5	3	2	AD		
104	77	female	6	.5	25	2	Carriers	e4e4	44	4	3	3	MCI	46	
106	81	female	6	.5	24	1	Non-carriers	e2e3	10	3	2	2	MCI	10	

110	80	male	16	.5	18	3	Non-carriers	e3e3	33	8	3	3	MCI	31	48
112	72	male	16	0	27	0	Non-carriers	e3e3	23	1	1	0	Normal		17
114	76	female	6	1	15	7	Carriers	e3e4	10	1	1	1	AD	3	
115	84	female	12	.5	26	2	Non-carriers	e3e3	18	6	3	3	MCI	18	
116	74	female	6	0	29	0	Non-carriers	e3e3	3	2	1	1	Normal		3
117	85	female	16	0	27	0	Carriers	e3e4	16	2	3	1	MCI		
119	63	male	18	0	30	0	Non-carriers	e3e3	0	0	0	1	Normal	0	0
120	80	female	6	.5	23	3	Non-carriers	e3e3	45	2	3	3	MCI	47	
121	88	male	16	.5	24	3	Non-carriers	e2e3	19	6	3	2	MCI	19	18
122	71	female	6	0	24	0	Non-carriers	e3e3	3	2	1	1	MCI		3
124	80	male	16	2	7	13	Non-carriers	e3e3	0	5	0	0	AD		0
125	81	female	0	.5	10	4	Non-carriers	e3e3	35	3	3	2	AD	29	
127	67	female	6	.5	22	2	Carriers	e4e4	0	2	1	1	MCI	0	0
128	80	female	16	0	27	0	Carriers	e3e4	8	0	2	1	AD	4	
130	81	female	12	1	21	6	Non-carriers	e2e3	16	5	3	2	AD		
131	74	female	16	.5	27	2	Non-carriers	e3e3	0	0	1	1	MCI	0	0
132	69	male	14	0	30	0	Non-carriers	e2e3	2	0	1	0	Normal	3	2
133	73	male	6	0	28	0	Non-carriers	e3e3	18	4	3	2	Normal		
134	72	male	18	.5	29	1	Carriers	e3e4	1	3	1	1	MCI	1	1
135	66	female	12	0	29	0	Non-carriers	e3e3	20	0	3	2	Normal	27	18
136	78	female	9	0	29	0	Non-carriers	e3e3	36	2	3	3	Normal	38	
137	66	female	14	0	29	0	Non-carriers	e3e3	8	4	2	1	MCI		
138	67	female	12	1	26	6	Non-carriers	e3e3	12	2	3	2	MCI	12	
139	81	male	18	0	30	0	Non-carriers	e3e3	8	2	2	1	Normal	11	
141	58	female	12	.5	30	0	Non-carriers	e3e3	4	0	0	0	MCI		4
142	90	female	0	1	10	5	Non-carriers	e3e3	6	2	2	1	AD		
143	75	female	6	.5	20	3	Non-carriers	e2e3	21	2	3	3	MCI		
144	82	female	6	1	13	5	Non-carriers	e3e3	10	6	1	1	AD		
145	61	male	12	0	29	0	Non-carriers	e2e3	34	0	2	2	MCI		31
147	68	male	6	0	28	0	Non-carriers	e3e3	5	4	2	1	MCI		5
148	61	female	12	0	30	0	Non-carriers	e3e3	9	1	1	0	MCI		
149	75	female	6	1	13	7	Carriers	e3e4	6	2	1	1	AD	4	
150	89	female	9	2	15	12	Carriers	e2e4	53	4	3	3	AD		58
151	88	male	12	0	30	0	Non-carriers	e3e3	22	2	2	1	MCI		
152	59	male	14	.5	27	2	Carriers	e3e4	6	2	1	1	MCI	6	
153	85	male	9	.5	14	4	Non-carriers	e3e3	2	4	2	0	MCI		2
154	80	female	6	.5	28	1	Non-carriers	e3e3	23	4	3	3	MCI	23	
155	72	female	6	1	22	5	Non-carriers	e3e3	1	4	2	1	MCI		1
157	82	male	0	1	4	5	Non-carriers	e3e3	32	4	3	3	MCI	29	35
158	78	female	6	1	11	9	Non-carriers	e3e3	9	2	2	1	AD		

160	82	female	12	.5	20	3	Non-carriers	e3e3	48	6	3	3	MCI	49	48
161	90	female	9	1	15	6	Non-carriers	e3e3	20	2	3	3	AD		
163	76	male	16	2	9	13	Non-carriers	e3e3	5	6	2	1	AD		5
164	75	male	9	0	30	0	Non-carriers	e3e3	3	2	2	1	Normal	11	3
165	79	female	3	1	12	7	Carriers	e3e4	2	4	1	0	AD		2
166	69	male	12	0	29	0	Non-carriers	e3e3	14	0	2	2	Normal	9	
169	66	male	6	.5	18	2	Non-carriers	e3e3	6	2	1	1	MCI	4	
170	83	female	0	1	7	6	Carriers	e3e4	36	4	3	3	AD		40
171	85	male	12	.5	24	2	Non-carriers	e3e3	24	2	2	2	MCI	33	24
173	73	male	6	.5	27	2	Carriers	e3e4	10	2	1	1	MCI	6	7
174	73	female	6	.5	16	4	Non-carriers	e3e3	6	3	2	1	MCI		
176	85	female	6	1	19	6	Carriers	e3e4	13	5	2	1	AD	9	14
177	52	male	9	.5	28	1	Non-carriers	e3e3	52	1	3	3	MCI		47
178	81	female	0	0	27	0	Non-carriers	e3e3	4	2	2	1	Normal		4
179	77	female	12	1	13	9	Carriers	e3e4	10	4	1	1	AD	4	7
180	74	female	16	0	30	0	Carriers	e3e4	10	0	2	0	Normal		
181	63	male	16	.5	30	1	Non-carriers	e3e3	55	2	3	3	MCI	55	58
338	84	male	1	.5	16	1	Non-carriers	e3e3	10	2	2	1	MCI	10	
630	72	female	6	1	13	7	Carriers	e3e4	6	3	1	1	AD		
803	74	female	16	1	18	6	Carriers	e3e4	19	2	1	1	AD		
833	78	female	0	.5	22	1	Non-carriers	e3e3	26	2	2	2	MCI		
836	69	female	2	2	6	10	Carriers	e3e4	24	3	0	1	AD		
855	75	female	16	1	19	6	Carriers	e3e4	19	1	1	0	AD		
868	82	female	3	.5	13	3	Carriers	e3e4	29	6	2	2	MCI		
874	82	male	12	1	11	8	Non-carriers	e3e3	17	2	1	1	AD		
875	82	female	14	1	15	7	Carriers	e3e4	12	4	1	1	AD		
876	89	female	7	.5	20	1	Non-carriers	e3e3	23	2	1	2	MCI	27	
880	82	female	9	0	30	0	Non-carriers	e3e3	6	0	1	1	Normal		
887	81	female	12	0	27	0	Non-carriers	e3e3	34	2	1	2	Normal	40	
888	85	female	12	.5	21	2	Non-carriers	e3e3	22	3	2	1	MCI	24	
901	84	male	9	.5	17	4	Carriers	e3e4	17	2	1	1	MCI	18	
911	78	female	9	.5	21	2	Non-carriers	e3e3	37	2	2	3	MCI		39
943	77	male	8	.5	28	1	Non-carriers	e3e3	1	1	0	1	MCI	2	1
945	73	male	9	0	25	0	Non-carriers	e3e3	3	0	0	0	Normal	0	3
948	66	male	12	.5	22	2	Non-carriers	e3e3	1	0	0	0	MCI		1
949	66	male	16	0	30	0	Carriers	e3e4	8	1	0	1	Normal		
963	71	male	16	0	27	0	Non-carriers	e3e3	27	6	3	3	Normal	31	22
1005	65	female	12	.5	25	2	Non-carriers	e3e3	14	1	1	1	MCI		
1006	85	male	14	.5	26	3	Non-carriers	e2e3	10	0	0	0	MCI		
1013	66	female	16	0	28	0	Non-carriers	e2e3	0	0	0	0	Normal		0

1037	72	male	6	.5	27	2	Non-carriers	e3e3	23	5	2	2	MCI		
1067	75	male	14	.5	25	2	Carriers	e3e4	12	2	1	1	MCI	10	
1075	81	female	0	.5	14	4	Non-carriers	e3e3	21	3	2	3	MCI		
1104	81	male	12	1	18	7	Carriers	e3e4	17	4	1	2	AD		
1112	86	male	6	2	11	13	Carriers	e3e4	16	4	1	1	AD		
1145	73	female	6	2	14	10	Non-carriers	e3e3	8	2	1	0	AD		
1158	74	female	4	1	10	5	Carriers	e3e4	34	3	3	3	AD		
1182	84	female	12	.5	28	1	Non-carriers	e3e3	14	2	1	2	MCI		
